# Improved base calling for the Illumina Genome Analyzer using machine learning strategies

**DOI:** 10.1186/gb-2009-10-8-r83

**Published:** 2009-08-14

**Authors:** Martin Kircher, Udo Stenzel, Janet Kelso

**Affiliations:** 1 Department of Evolutionary Genetics, Max Planck Institute for Evolutionary Anthropology, Deutscher Platz, 04103 Leipzig, Germany

## Abstract

The Illumina Genome Analyzer generates millions of short sequencing reads. We present Ibis (Improved base identification system), an accurate, fast and easy-to-use base caller that significantly reduces the error rate and increases the output of usable reads. Ibis is faster and more robust with respect to chemistry and technology than other publicly available packages. Ibis is freely available under the GPL from .

## Abstract 2

Ibis is an accurate, fast and easy-to-use base caller for the Illumina Genome Analyzer that reduces error rates and increases output of usable reads.

## Rationale

Recent advances in high-throughput sequencing have revolutionized genomics, making it possible for even single research groups to generate large amounts of sequence data very rapidly and at substantially lower costs than traditional Sanger sequencing. This puts the ability to perform deep transcriptome sequencing and transcript quantification, whole genome sequencing and resequencing into the hands of many more researchers. However, while cost and time have been greatly reduced, the error profiles of next-generation platforms differ significantly to those of previous approaches. By addressing this issue, the number of sequences and the quality of the data can be optimized.

The Illumina Genome Analyzer is based on parallel, fluorescence-based readout of millions of immobilized sequences that are iteratively sequenced using reversible terminator chemistry [1]. In brief, up to eight DNA libraries are hybridized to an eight-lane flow cell. In each of the lanes, single-stranded library molecules hybridize to complementary oligos that are covalently bound to the flow cell surface. Using the double stranded duplex, the reverse strand of each library molecule is synthesized and the now covalently bound molecule is then further amplified in a process called bridge amplification. This generates clusters each containing more than 1,000 copies of the starting molecule. One strand is then selectively removed, free ends are subsequently blocked and a sequencing primer is annealed onto the adapter sequences of the cluster molecules.

Starting from the sequencing primers, 3' terminated and fluorescence-labeled nucleotides are incorporated using a modified polymerase. Base incorporation ceases after the addition of a single base due to the 3' termination of the incorporated nucleotides. The fluorophores attached to the nucleotides are illuminated using a red and a green laser, and imaged through different filters, yielding four images per tile. The number of tiles varies; for Genome Analyzer I it is typically 300 tiles per lane, for Genome Analyzer II it is 100 tiles per lane. After an imaging cycle, the fluorescent labels as well as the 3' terminators are chemically removed and the next incorporation cycle is started. Incorporation and imaging cycles are repeated up to a designated number of cycles, defining the read length for all clusters.

After sequencing, images are analyzed and intensities extracted for each cluster. The Illumina base caller, Bustard, has to handle two effects of the four intensity values extracted for each cycle and cluster: first, a strong correlation of the A and C intensities as well as of the G and T intensities due to similar emission spectra of the fluorophores and limited separation by the filters used; and second, dependence of the signal for a specific cycle on the signal of the cycles before and after, known as phasing and pre-phasing, respectively. Phasing and pre-phasing are caused by incomplete removal of the 3' terminators and fluorophores, sequences in the cluster missing an incorporation cycle, as well as by the incorporation of nucleotides without effective 3' terminators. Phasing and pre-phasing cause the extracted intensities for a specific cycle to consist of the signal of the current cycle as well as noise from the preceding and following cycles. As the number of cycles increases, the fraction of sequences per cluster affected by phasing increases, hampering the identification of the correct base.

Technical improvements in the filters and camera of the Genome Analyzer II have helped with distinguishing the A and C as well as G and T fluorophores. Phasing and pre-phasing was addressed by an improvement of the sequencing chemistry kit that became publically available in the late summer of 2008. This new sequencing chemistry preparation (order numbers FC-204-20xx) reduced the phasing rates determined by Bustard from, on average, 0.8% per cycle to 0.5%, and pre-phasing from 0.6% to 0.4% per cycle. In 2009, Illumina introduced a new chemistry (FC-103-300x) and further updates are expected within the year. Both improvements reduced the overall error rate and allow more sequencing cycles. Here, we present an improvement for the base calling on the Illumina Genome Analyzer platform that can be used for all versions of the Genome Analyzer platforms and chemistries to further decrease the overall error rate.

Two publications [2,3] addressed the base calling of the Illumina platform, both using statistical learners trained on sequences called by the standard base caller, Bustard. Statistical learners, also called machine-learning approaches, describe a wide range of mathematical models and algorithms used to extract patterns and rules from huge data sets. In general, statistical learning can facilitate a better understanding of the basics underlying data or can be applied for predicting both qualitative (that is, discrete labels) and quantitative descriptors (that is, values out of a continuous range) from data. In this context, base calling can be seen as predicting discrete labels, finding the correct nucleotide label given the intensity values observed for a specific cycle (that is, a four-class classification problem).

Erlich *et al*. [2] published AltaCyclic, the first machine-learning based approach to base calling for the Genome Analyzer. Their approach applies support vector machines (SVMs) trained for each individual cycle. Rolexa [3], a base caller for the statistical software package R [4], applies Gaussian mixture models, similar to the approach used by Cokus *et al*. [5] for the analysis of bisulphite sequencing data. The two base callers differ further in that Rolexa generates ambiguity codes for potential erroneous base calls, while AltaCyclic produces unambiguous bases with quality scores.

We present Ibis (Improved base identification system), an accurate, fast and easy-to-use base caller for the Illumina sequencing system, which aims to significantly reduce the error rate and increase the output of usable reads. Our goal is to provide sequences with a lower number of base calling errors and better quality scores with each base. This will facilitate quality filtering of the data, sequence read mapping, *de novo *assembly and further data analysis like single nucleotide polymorphism (SNP) calling.

## Results

### Intensity files and the Illumina standard base caller

Briefly described before, the Genome Analyzer takes four images per tile and cycle during the sequencing run. The image analysis software of the IPAR (Integrated Primary Analysis and Reporting) machine, the RTA (Real Time Analysis) software or the Firecrest program of the Analysis Pipeline registers the four images, which are slightly scaled and shifted due to the different filters used, and identifies the clusters in the images. The images are then further registered between cycles and the intensity values extracted from the four images for each of the clusters identified. This results in four floating point numbers per clusters and cycle. A cluster is identified by the quadruple of lane number, tile number and x-y coordinates of the cluster in the superimposed reference image. Depending on the image analysis software (IPAR, Firecrest, RTA) the created output files vary, but otherwise provide the same input for the base calling process.

As shown previously [2,3], the intensities of the A and C channels are highly correlated as are those of the G and T channels due to similar emission spectra of the fluorophores used for A and C and G and T. In order to separate these channels and normalize their individual intensities, the Illumina base caller (Bustard) uses a so-called crosstalk matrix estimated from the first or second imaging cycle. This estimate, however, is based on the assumption that the four nucleotides are almost equally frequent at each sequence position in the library being sequenced. If this assumption is violated, the inaccurate estimates can lead to incorrect base calling. To prevent this, the crosstalk matrix is commonly estimated using a control lane in which a variant of PhiX 174 (GC content of 44.7%) is sequenced. This PhiX variant RF1 also allows for different quality control measures, and is therefore widely used as control lane to track run quality and to facilitate base calling.

Bustard estimates the phasing and pre-phasing as two channel-independent parameters from the increasing correlation of intensities in the first few cycles of the sequencing run. Using the crosstalk matrix and the two phasing parameters, Bustard creates corrected intensity values and calls the base with the highest corrected intensity for each cluster and cycle. In the case of equal intensity values or small intensity differences an 'N' is called. Further, a trust value is assigned to each intensity value. If a FastQ file is created, the trust value of the called base is transformed to an ASCII character (using an offset of 64).

**Figure 1:**
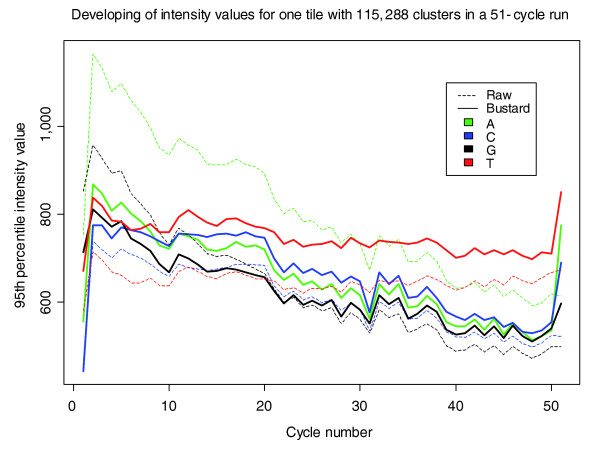
**Intensity values for one tile of a 51-cycle PhiX 174 RF1 run before and after correction by Bustard**. On this tile 115,288 clusters were identified by the image analysis software Firecrest. Shown are the 95^th ^percentile for the signal intensities in each channel and cycle. The raw intensities are shown with dashed lines, the intensities after transformation by Bustard are shown with solid lines. Intensities for A, C, and G decline over the run while the intensities for T stay nearly constant. Both effects can be explained by degradation of the fluorophores or non-reversible termination of sequences over the run as well as the accumulation of T fluorophores on the synthesized strand. Intensities for the first cycle are lower than in other cycles due to dimming and bleaching caused by longer handling times before imaging of the first cycle. Corrected intensities for the last and first cycle do not follow the normal trend, since full phasing correction cannot be applied.

**Figure 2:**
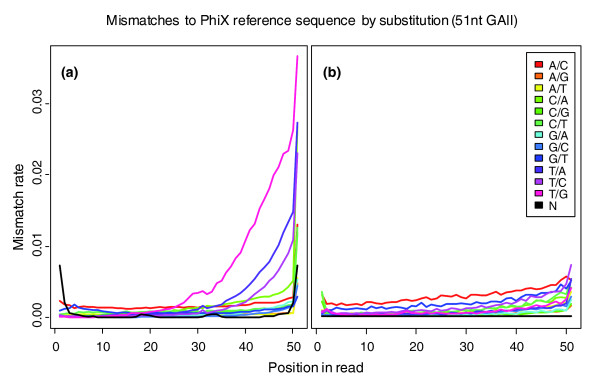
**Analysis of mismatches**. Analysis of mismatches seen for **(a) **Bustard raw reads and **(b) **Ibis raw reads of a lane with 11,478,043 PhiX 174 RF1 raw reads sequenced with 51 cycles and mapped to the corresponding reference genome allowing up to 5 mismatches (including N characters). For Bustard 9,110,666 (79.4%) raw reads can be mapped, and for Ibis 9,695,354 (84.5%) raw reads. The sequencing error, measured as the mismatch rate, increases with cycle number. For Bustard, starting around cycle 25, guanine is mistaken as thymine. In later cycles adenosine and cytosine are also mistaken as thymine, due to increasing T accumulation. The error rate of the last base is especially high due to incomplete phasing correction. The patterns of specific base mismatches are not observed when Ibis is used.

The Bustard base calling process described here is based on two additional assumptions: first, that the crosstalk matrix can be considered constant over the run; and second, that phasing affects all nucleotides in the same way. Erlich *et al*. [2] have previously shown that this first assumption is violated. Another argument for this is the commonly observed decrease in intensities over the course of the run (Figure 1). This is likely to be a result of degradation of the fluorophores, or the effect of a decreasing number of sequences being elongated in each cluster when nucleotides for which the termination cannot be removed are incorporated (as also suggested by Erlich *et al*. [2]). Further, we see that phasing does not affect all nucleotides equally. With the chemistries FC-104-100x or FC-204-20xx, the fluorophores used for thymine show a lower removal rate after treatment with TCEP (tris-(2-carboxyethyl)-phosphine) [1] and accumulate over the sequencing run (T accumulation; Figures 1 and 2).

The effects of crosstalk, declining intensities, pre-phasing and phasing, as well as T accumulation complicate the identification of the correct base, especially in later sequencing cycles. When mapping raw reads of PhiX 174 RF1 sequenced with 51 cycles, 79.4% map to the corresponding reference genome allowing up to 5 mismatches. Only 39.8% map without any mismatches. Analyzing the different types of mismatches, we observe a non-random distribution (Figure 2a). Starting around cycle 25, guanine is increasingly confused with thymine (illuminated using the same laser); in later cycles adenosine and cytosine show also a high rate of erroneous thymine calls due to increasing T accumulation. The error rate of the first base is especially high due to the higher handling time when starting the sequencing run (for example, focusing and first cycle report); the last base is especially high due to the inability to correct phasing completely.

### Statistical learner for Illumina base calling

When designing a base caller that can cope with the cycle-dependent problems discussed above, we considered constructing a more complex model of the sequencing chemistry than is currently available in Bustard - including T accumulation, declining intensities and the specific characteristics of the first and last cycle. All currently available base callers follow this general approach, although the complexity of the model and the modeled parameters differ. However, this approach has two major disadvantages. First, building a correct model for the Illumina sequencing platform requires a deep understanding of the causes for sequencing error and is likely to be incomplete. Secondly, a sufficiently complex model will depend on the chemistry or platform version used and has to be adjusted when either one changes. We instead chose to estimate the sequencing chemistry model as a parameter directly from the data using statistical learners and a training data set derived from the Bustard output.

Previous approaches [2,3] corrected raw intensities prior to the application of the statistical learner and used only the intensities of one cycle as input. This causes these approaches to be highly dependent on a correct modeling, or at least very good modeling, of the sequencing process. We bypassed this problem by directly basing our training on the raw cluster intensities. To identify the correct number of cycles as input for the statistical learner, we first simulated clusters of a thousand sequences and the fluorophore attachment over several sequencing cycles using the model of the sequencing process described above with pre-phasing, phasing and T accumulation. We used a symmetric phasing and pre-phasing rate of 0.4% and a T accumulation rate of 3.8% per cycle (for a detailed description see Additional data file 1).

Simulating up to 150 cycles, we observed that, for a typical read length of 50 cycles, 59.5% of the fluorophores reflect the current cycle, 17.4% are exactly one cycle behind and the same fraction is one cycle ahead, and 33.9% of the measured cluster intensity is caused by T accumulation. Even after 150 cycles, 85.1% of the fluorophores account for the previous, the current or the next base to be sequenced (Figure S2 and Table S2 in Additional data file 1). From this simulation, we conclude that most of the signal to be captured by a statistical learner is contained in the raw intensities of the previous, the current and the next cycle.

We therefore implemented a base caller with SVM classifiers for each cycle that have the intensity values of the current cycle and its two neighbors as input. The exceptions are the first and last cycle, where we can only include one of the neighbors. For the SVM classifiers of each cycle, we use a computationally fast implementation of multiclass SVMs with polynomial kernels, called SVM^multiclass ^[6]. A putative training data set is created by aligning the Bustard raw reads with mismatches for a fraction of the tiles to an appropriate reference sequence (for example, PhiX 174 RF1) using SOAP [7]. We keep half of this data set as a test data set and use the other half for training the classifiers separating all four nucleotide classes (A, C, G, and T) in each cycle.

We verify the result of the training by using the test data set with the trained models and comparing the predicted labels with the ones obtained from the reference sequence. Evaluating this information, we can also estimate parameters for calculating a quality score for each called base given the class assignment and the distances to the classification/decision boundary reported by SVM^multiclass^. Based on this measure, we use the density distributions for the four distances to the decision boundary seen for each correct class label (16 in total, each following a normal distribution based on Shapiro Wilk Normality test). Given the four distances *d*_*Z *_(*z ∈ {A, C, G, T}*) and the parameters estimated from the test data set, we define the likelihood of the called base being wrong as:



We extended the SVM^multiclass ^C/C++ package by routines that are able to handle several classifiers in parallel for the individual cycles, parse Firecrest, RTA and IPAR output files, calculate quality scores and create Sanger-like (using an offset of 33) FastQ output files. Applying this approach to the lane shown in Figure 2a increases the number of perfectly mapped sequences from 39.8% to 60.2% (from 4,564,039 to 6,908,856) and shows an error profile of all mapped sequences (9,695,354 out of 11,478,043) as depicted in Figure 2b.

## Discussion

### Other systems for base calling

Applying statistical learning for the base calling of Illumina sequences is not novel. However, Ibis differs significantly in its concept and its performance. AltaCyclic [2] uses a model of phasing/pre-phasing, fluorescent decay and cycle-dependent crosstalk to correct raw intensities before classification, using SVM classifiers trained individually for every cycle. The AltaCyclic model does not include base-specific phasing parameters and, therefore, cannot correct raw intensities for the observed T accumulation effect. Similarly, the Rolexa package [3] corrects the raw intensities prior to the application of Gaussian mixture models as classifiers. In contrast to the models of sequencing chemistry implemented in AltaCyclic, Rolexa models only crosstalk and single-parameter phasing (pre-phasing is not modeled). In contrast to AltaCyclic, Bustard and Ibis, Rolexa applies a transformation to the intensities within each tile to correct for differences in the illumination of clusters. Further Rolexa uses IUPAC ambiguity codes to encode uncertainty in base calling, while AltaCyclic, Bustard and Ibis try to call one correct base and reflect the associated uncertainty in the quality scores. The latter approach is superior when the sequences are mapped and analyzed with software that is unable to handle ambiguity codes (like most currently available fast mappers or SNP calling software). Unlike AltaCyclic and Bustard, Ibis does not call an 'N' character for low quality bases, as the most likely base can still be informative and the uncertainty is already captured in the quality score.

### Performance test

The difference in introducing IUPAC ambiguity codes complicates the direct comparison of AltaCyclic, Bustard, Ibis and Rolexa. We therefore forced Rolexa to call sequences without using ambiguity codes, and we specifically consider 'N' characters for a direct comparison. We tested the performance of the four different base callers on five data sets of which we present two data sets in the main text and the others in Additional data file 1: a 26 cycle Genome Analyzer I run of which we analyzed the PhiX control lane (A1) and one lane with human shotgun sequences (A2); and a 51 cycle Genome Analyzer II run of which we only analyzed the PhiX control lane (B). For lanes A1 and B we mapped all control lane sequences to the PhiX reference sequence allowing up to five mismatches but no gaps using SOAP v1.11 [7]. For the lane with human shotgun sequences (A2), we mapped the sequences to the human reference genome (hg18/NCBI Build 36.1) allowing five mismatches without any gaps. However, for this data set we restricted the further analysis to sequences mapping with at most two mismatches to reduce the number of false positive placements expected when using a genome with almost three billion bases and short reads.

**Figure 3:**
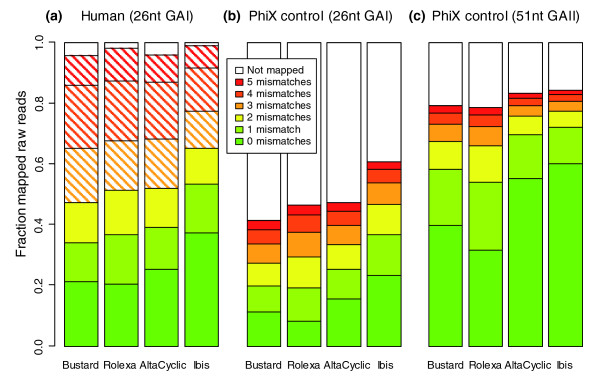
**Fraction of mapped reads and corresponding number of mismatches for the three tested lanes**. **(a) **The result for one lane of human shot gun sequence analyzed on a 26 cycle Genome Analyzer I run (A1); **(b) **the PhiX control lane of the very same 26 cycle Genome Analyzer I run (A2); **(c) **the PhiX control lane of a 51 cycle Genome Analyzer II (B). The raw sequences of all three lanes were mapped to the corresponding reference genome (hg18/NCBI Build 36.1 and PhiX 174 RF1) with up to five mismatches but no gaps using SOAP v1.11. For A1, further analyses were restricted to sequences mapping with at most two mismatches to reduce the number false positive placements expected when mapping short reads to a large genome sequence.

The fraction of mapped raw reads and corresponding number of mismatches for the three lanes is shown in Figure 3. The number of correct reads when using Ibis compared to Bustard increased about 2.1-fold in A1 (11.3% to 23.4%), 1.8-fold in A2 (21.2% to 37.4%), and 1.5-fold in C (39.8% to 60.2%). When comparing the error profiles in B (Figure 2), we see that Ibis was able to correct for the T accumulation pattern seen in Figure 1. Assuming that all reads belong to the corresponding reference, we can give a (lower) estimate of the error rate in the run (assuming the remaining reads would be matched when allowing one more mismatch). For A1 these are 15.2%, 16.4%, 12.3% and 16.0% for AltaCyclic, Bustard, Ibis and Rolexa, respectively. For A2 (assuming to match the rest with 3 mismatches) these are 7.1%, 7.6%, 5.5%, and 7.4%. In the third lane (B), the 51 cycle PhiX control, the error rate is much lower (due to the better quality of the run as well as technical improvements of the Genome Analyzer II instrument and chemistry); the rates for AltaCyclic, Bustard, Ibis and Rolexa are 3.0%, 4.0%, 2.8% and 4.3%, respectively. The development of the mismatch rates per cycle observed in the mapping for each of the three other data sets is available in Additional data file 1. Summarizing the results of all five data sets, Ibis outperforms the other programs in base calling accuracy. Similarly, we see improved performance of Ibis over other base callers when comparing the performance of Bustard, AltaCyclic and Ibis for longer Genome Analyzer II runs (76, 77 and 101 cycles) using different chemistries (Figures S6, S7 and S8 in Additional data file 1, respectively).

**Figure 4:**
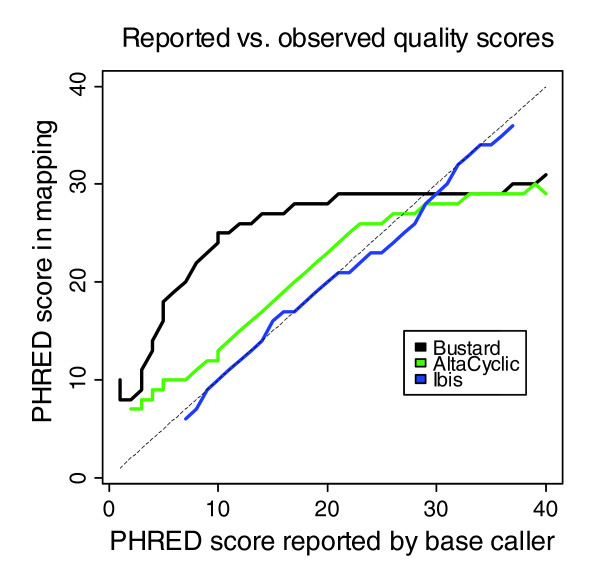
**Comparison of quality scores for the 51 cycle PhiX control lane data**. Quality scores reported by Bustard, AltaCyclic and Ibis are compared in PHRED scale. For all three base callers, we considered only quality scores reported with 100,000 and more observations. Calculating the deviation from the optimal line, Bustard has a root mean square deviation of 84.9, AltaCyclic of 19.3 and Ibis of 0.9.

For B, we also compared the quality scores reported by Bustard, Alta-Cyclic and Ibis. While Ibis provides PHRED-like quality scores, Bustard and AltaCyclic use the Illumina-specific encoding of quality scores with a different offset and a different formula (Illumina Analysis Pipeline 1.0 and earlier versions). Therefore, quality scores from AltaCyclic and Bustard were converted to PHRED-like quality scores and compared in PHRED scale. The results are available in Figure 4. When measuring the deviation from the optimal line, Bustard has a root mean square deviation of 84.9, AltaCyclic of 19.3 and Ibis of 0.9. Hence, Ibis provides useful quality scores for further analyses.

As is the case for Bustard, AltaCyclic and Rolexa, the results of A1 and A2 support the assumption that training on the PhiX extends well to the prediction of other lanes using the same estimated models. To further verify this, we also tested with several other sequencing runs (Figures S7 and S8 in Additional data file 1) and did a specific test for overtraining (for example, learning base composition) and undertraining on PhiX for another 51 cycle run (data not shown). We trained several models from the PhiX lane using different numbers of tiles for training and predicted with the resulting models the PhiX lane as well as one of the other lanes. We then examined the number of sequences mapped to the two different reference genomes and the number of mismatches observed. We found no evidence for overtraining; however, we did observe undertraining affecting the prediction of both lanes. In our test, undertraining resulted in 3 to 5% fewer perfect reads and only up to 1% less mappable raw reads than obtained when using at least 1,000,000 sequences for training (about 10 to 15 tiles).

To compare the computational resources required for base calling, we measured the time for training and predicting the 51 cycle PhiX control lane (B) with each of the base callers. Base calling this lane using Bustard on an eight core system took 50 minutes (including estimation of crosstalk and phasing parameters) and created the input needed for all three other base callers. AltaCyclic needs a cluster system to run. Using about 80 cores of our cluster system, AltaCyclic took about 5.5 hours for the parameter estimation and 40 minutes for the base calling. On an eight core system these would correspond to at most 61 hours in total. Running Rolexa on an eight core machine took 17.5 hours. Ibis took 89 minutes for parameter estimation and 12 minutes for prediction, in total about 1.7 hours. In other words, using Ibis one has to invest three times more time for base calling, for Rolexa 21-fold more time and for AltaCyclic 73-fold more time compared to Bustard.

Ibis is not dependent on the inclusion of the PhiX control lane. In the case of resequencing projects or projects where some subset of the sequences generated comes from a previously characterized genome (for example, mitochondrial sequences) it is possible to use these data as a training dataset for Ibis. We have shown that it is possible to use the mitochondrial sequences generated as part of a shotgun sequencing experiment as an alternative training set (Figures S7 and S8 in Additional data file 1). Further, the raw Bustard output can be used as training data in cases where there is no reference set available (Additional data file 1), although the reduction in error rate is less than can be obtained when a reference is available.

### Further applications

Even though Ibis was originally developed to handle the T accumulation in a sequencing chemistry that has been replaced by a new version (FC-103-300x), its application is not limited to the reprocessing of data created with the older chemistries (FC-104-100x or FC-204-20xx). We have shown that Ibis improves the output of sequencing runs from the Genome Analyzer I, which due to their short read length are barely affected by T accumulation but by a generally lower image and sequencing quality. The reason is the sequencing model independent training process of Ibis, which only relies on the assumption that the vast majority of the signal needed for base calling is captured by the intensity values of the previous, the current and the next cycle. When using Ibis on data from experiments with the new sequencing chemistry (data shown in Additional data file 1), we also observe an improvement in base calling accuracy over Bustard. We are confident, therefore, that there is a benefit in investing a little more computational time in re-base-calling sequencing runs of all chemistry and Genome Analyzer versions.

## Conclusions

We were able to show that Ibis improves base calling accuracy compared to other Illumina base callers. Our approach is unique in that the causes of sequencing error are not modeled separately, but captured by incorporating neighboring signals in the statistical learning procedure. Due to this design, Ibis works on a wide range of different sequencing chemistries and platform versions. The performance of Ibis on standard hardware is significantly better than existing base callers, enabling it to be run by research laboratories without access to large computational clusters. The increase in mappable sequences, without ambiguity codes and improved quality scores, enables direct use of the sequences in other software packages. Ongoing development of the chemistry and hardware of the Illumina next-generation sequencing platforms will undoubtedly mean increases in read length and quality. We believe that our general approach will be applicable to further generations of the Illumina platform and provide improvements in sequence quality and confidence measures required for applications such as SNP calling and assembly.

## Materials and methods

### Sequencing

Sequencing was performed on Genome Analyzer I and Genome Analyzer II machines. Where not stated otherwise, standard protocols and kits available from Illumina, Inc. [1] were used for library preparation and sequencing. In the case of the runs with 51 and 77 bases, shorter sequencing protocol files for Genome Analyzer II available from Illumina, Inc. [1] were extended by duplication of cycles up to the designated number of cycles. In the case of the 51 cycle run, one 36 cycle sequencing kit (FC-104-1003) was prepared to yield the volume needed for 51 cycles. For the 77 cycle run, two 36 cycle sequencing kits (FC-204-2036) were pooled to yield the volume needed, and for the 76 cycle run two 36 cycle sequencing kits (FC-103-3003) were used. For the 101 cycle run, three 36 cycle sequencing kits (FC-103-3003) and a new polymerase provided by Illumina within an early access program were used.

### Ibis base caller

Ibis applies the SVM^multiclass ^package by Thorsten Joachims, which is an implementation of multi-class SVMs described by Crammer *et al*. [8]. As described in the main text, we added routines for processing IPAR, Firecrest and RTA files, extracting training and test data sets, training models for each individual cycle, fitting an error model to the test data and applying the trained models to the intensity files of each individual tile of the sequencing run. Ibis has been tested on Illumina pipeline versions 0.3.0, 1.0, 1.3.2 and 1.4.0.

The training and test data sets are created based on mapping sequences extracted from the Bustard base caller (for the 26 cycle and 51 cycle data sets presented, Bustard v1.9.5; for the 77 cycle data set, Bustard v1.3.2; and for the 76 and 101 cycle runs, Bustard v1.4.0) to a reference genome using SOAP v1.11 [7]. For each mapped sequence, we consider the sequence of the reference to be the correct one. For each cycle/position of the read, one SVM multiclass model is trained using svm_multiclass_learn. After training, the misclassification rate of each model and class is assessed using the test data set and svm_multiclass_classify. The models are then applied to the data of the complete run using a custom C++ interface to the SVM^multiclass ^package. For each cluster in the intensity files an entry in a FastQ file is created, containing the sequence and PHRED-like quality scores [9] in the Sanger encoding (with a quality score offset of 33).

### Other base callers

In addition to the Illumina standard base caller Bustard v1.9.5, we used AltaCyclic v0.1.1 and Rolexa v1.1.6 (with R v2.8.0). Standard parameters were used where applicable. For Rolexa three parameters were set to turn off ambiguity codes: Rolexa.env$HThresholds <- c(2.0,2.0,2.0); Rolexa.env$IThresholds <- (log2(41:nrcycles/6)); Rolexa.env$iupac <- c("A", "C", "G", "T", "N", "N", "N", "N", "N", "N", "N", "N", "N", "N").

AltaCyclic and Bustard quality scores were converted to PHRED-like quality scores by back calculating the probably *P *= 1/(1 + pow(10, Q_S_/10)) from the reported quality scores and PHRED log transformation Q_P _= ROUND(-10*log_10_(p)).

## Abbreviations

GA: Genome Analyzer; Ibis: Improved base identification system; IPAR: Integrated Primary Analysis and Reporting; nt: nucleotide; RTA: Real Time Analysis; SNP: single nucleotide polymorphism; SVM: support vector machine.

## Authors' contributions

Programming and analyses were performed by MK with input by US and JK. The manuscript was written by MK and JK. All authors read and approved the final manuscript.

## Additional data files

The following additional data are available with the online version of this paper: a PDF document containing all additional supplementary figures (Figures S1 to S8) and Tables (Tables S1 to S2) (Additional data file 1).

## Supplementary Material

Additional data file 1 Figures S1 to S8 and Tables S1 to S2. Click here for file
