# Supplementary material for: Improved base calling for the Illumina Genome Analyzer using machine learning strategies
Source: Genome Biol. 2009 Aug 14;10(8):R83. doi: 10.1186/gb-2009-10-8-r83 (PMC2745764; doi:10.1186/gb-2009-10-8-r83)
Supplement: Additional data file 1 — Figures S1 to S8 and Tables S1 to S2. [file gb-2009-10-8-r83-S1.pdf]

Supplementary information for "Improved base calling for the Illumina Genome Analyzer using machine learning strategies"

Martin Kircher, Udo Stenzel, Janet Kelso

Department of Evolutionary Genetics, Max Planck Institute for Evolutionary Anthropology, Deutscher Platz 6, 04103 Leipzig, Germany

1. Simulating phasing, pre-phasing and T accumulation

To identify the correct number of cycles as input for the statistical learner, we simulated 10,000 clusters, each with 1,000 identical sequences. The 10,000 sequences were created randomly using the GC content of PhiX (44.7%) as a guide. We used a model with pre-phasing, phasing, T accumulation and simulated the fluorophores attached over 150 sequencing cycles.

For each cycle, we determined the number of fluorophores attached to the sequences of the cluster and calculated the fraction of fluorophores representing the current cycle, the previous cycle and the next cycle, as well as representing cycles more than one ahead and more than one behind. Further, we calculated the fraction of fluorophores attached due to T accumulation. The results were averaged over all sequences.

For phasing and pre-phasing parameters, we checked the values reported by Bustard (available in the params XML files of the Bustard subfolder of a run) for the last 10 runs done on our Genome Analyzer II sequencers (see table S1 on the right). While some runs seem to have balanced phasing and pre-phasing values, others showed higher phasing values. We picked the mean of the pre-phasing values and simulated symmetrical phasing/pre-phasing with a rate of 0.4% per cycle. Our simulation of the phasing process follows the recurrence:

Base case :

$$f_0(0) = 100\,\%, \quad f_{p\neq 0}(0) = 0\%, \quad f_0(c \neq 0) = 0\%$$

Recurrence :

$$f_{p\in\{1,\dots,l\}}(c) = \begin{aligned} & f_{p-1}(c-1) \\ & + \left(f_{p-2}(c-1) - f_{p-1}(c-1)\right)\cdot p_{pre} \\ & + \left(f_p(c-1) - f_{p-1}(c-1))\right)\cdot p \end{aligned}$$

Run	Phasing	Pre-phasing
**1**	0.0072	0.0049
**2**	0.0053	0.0053
**3**	0.0039	0.0038
**4**	0.0039	0.0038
**5**	0.0044	0.0035
**6**	0.0043	0.0042
**7**	0.0062	0.0043
**8**	0.0070	0.0033
**9**	0.0048	0.0030
**10**	0.0052	0.0036
Mean	0.0052	0.0040
Std deviation	0.0012	0.0007
Median	0.0050	0.0038

Table S1: Phasing and pre-phasing parameter estimates from Bustard for a selection of 10 runs done on our Genome Analyzer II sequencers.

T accumulation was estimated from the 51 cycle run presented as one of the PhiX test data sets and simulated as additional fluorophores with cycle information sticking on the clusters. From one tile (number 25 of this run) with 115,288 clusters we extracted the 5th percentile of the raw intensities in each of the 51 cycles. Due to the effects of bleaching and dimming in the first cycle (as a result of longer handling times), we excluded the first cycle from the analysis. We then normalized the values for each channel (A, C, G and T) separately, so that the 5th percentile raw intensity value of the second cycle is 100 in each of the four channels. As phasing and pre-

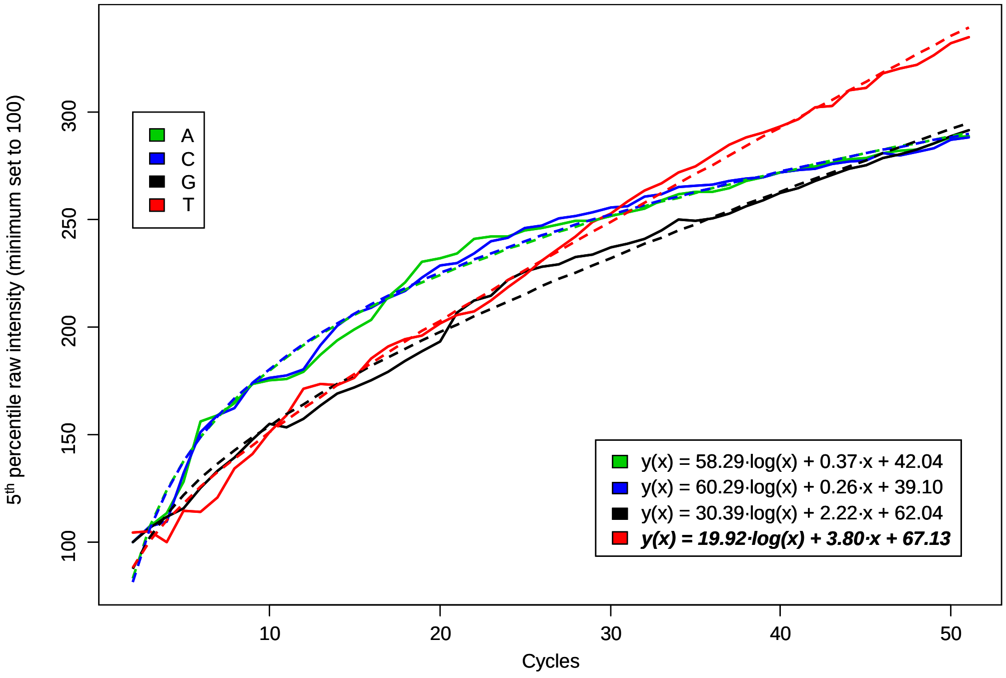

Figure S1: Development of the 5th percentile of the raw intensities over 50 cycles. Due to the bleaching/dimming effect of the first cycle, the first of 51 cycles has been excluded. The remaining values have normalized within in each channel (A, C, G and T) separately, so that the 5th percentile raw intensity were of the second cycle is 100. We fitted a function consisting of a linear (describing the accumulation of fluorophores) and a logarithmic part (describing noise increase due to phasing). For A and C the linear part does not significantly increase the quality of the fit (AIC). The linear effect is strongest in the T channel and accounts for an increase of 3.8% per cycle; the effect in the G channel is probably also caused by the T channel - due to the read out with the same laser (cross-talk).

phasing increases, the background noise in each cycle increases. We therefore expect to see an exponential increase of the background noise measured with the 5th percentile. We fitted a logarithmic function (noise = a·log(cycle) + n; inverse of the exponential function) to the values extracted. We got a very good fit for A and C, but G and T seemed to miss another linear factor: the described T accumulation. We therefore fitted noise = a·log(cycle) + b·cycle + n, resulting in the fits shown in figure S1. With the linear factor the function fits G and T better, for A and C there is no significant increase with respect to the Akaike information criterion (AIC). Due to cross-talk the linear effect observed for G may be completely caused by the accumulation of T fluorophores. Together with the observation of the base substitution patterns observed from Bustard, we therefore only modeled fluorophore accumulation of Ts, with a rate estimated from the fit with 3.8% per cycle. A table with the results of the simulation is available at the end of this document as table S2, a visual representation is available as figure S2.

2. Mismatch rates in short GA I data sets and data sets with T accumulation

We tested the performance of four different base callers (Bustard, Rolexa, AltaCyclic and Ibis) on two sequencing runs. The first being a 26 cycle Genome Analyzer I run of which we analyzed the PhiX control lane (figure S3) and one lane with human shot gun sequences (figure S4); and the second being a 51 cycle Genome Analyzer II run (FC-104-100x) of which we analyzed the PhiX control lane (figure S5). Further, we show a comparison of the three base callers on the PhiX control lane of a 77 cycle run, which was created using the FC-204-20xx chemistry. This

comparison does not include the Rolexa base caller as Rolexa crashes when processing these longer reads. For the 77 cycle run we show the base miscalls by substitution for each of the base callers in figure S6. The average error rates and the development of the error rate reported in the figure are based on reads mapped from the Bustard results and each of the base callers. We mapped the raw control lane sequences to the PhiX reference sequence allowing up to 5 mismatches but no gaps using SOAP v1.11. For the analysis of the SOAP output, we considered Ns in the sequence as mismatches to the reference and reinforced the 5 mismatch cutoff. For the lane with human shot gun sequences, we mapped the sequences to the human reference (hg18/NCBI Build 36.1) genome also allowing 5 mismatches without any gaps. However, we restricted the later analysis of the mismatch rates to sequences mapping with at most two mismatches to reduce the number false positive placements of short reads expected for a genome with almost three billion bases.

For the 77 cycle data set, we also created a training data set directly from the Bustard raw sequences without using a reference sequence. In this case we took about 3.7 million Bustard reads with at most three Ns as training data. This procedure can be considered as the 'last resort' for using Ibis in cases where no reference sequence is available for creating a better training data set. We see a reduction of the error rate by about 20% compared to Bustard and are able to map about 10% more reads with the same number of mismatches allowed (figure S6). This is, as expected, inferior to the results obtained using a reference (in which error rate reduction is more than 70% and 52% more reads are mapped). However, even without the reference the base calling using Ibis provides a considerable improvement compared to the Bustard base caller.

3. Mismatch rates in recent chemistries and without PhiX control lanes

Recently, Illumina released a new chemistry version (FC-103-300x) in which the T accumulation effect can no longer be observed. This was achieved by replacing the cleavage reagent used in the previous generation chemistry. Tests of a new polymerase used for the base incorporation are being completed and a new chemistry including this new polymerase is expected to be released soon. This new polymerase is intended to reduce the pre-phasing effect and therefore allow for longer reads. To show that the application of Ibis extends to these new chemistries, we show here a 76nt cycle run created with the FC-103-300x chemistry (figure S7) and a 101 cycle run (figure S8) that uses the new polymerase provided by Illumina (obtained within an early access program). In the figures, we show the base miscalls by substitution as well as the fraction of reads mapped for each base caller. These numbers were obtained by mapping the raw control lane sequences to the PhiX reference sequence allowing up to 5 mismatches but no gaps using SOAP v1.11. Again we considered Ns in the sequence as mismatches to the reference and reinforced the 5 mismatch cutoff before further analysis. Error rate estimates are based on reads mapped with Bustard and each of the base callers.

We compare the results obtained for the Illumina base caller (Bustard), AltaCyclic and our base caller (Ibis). We also included results for training Ibis without a reference, which again performed considerably better than Bustard (6%/16% more reads mapped and a reduction of the error rate by 28%/24% respectively). In addition, for these two runs we show that it is possible to use mitochondrial reads from shotgun experiments as an alternative way to create a training data set. In the first example (76 cycle run), about 50,000 sequences extracted from only one lane and in the second example (101 cycle run) 1.8 million human mitochondrial sequences extracted from several lanes of the run were used. In both cases, the results for using the PhiX control versus using the mitochondrial reads as input for training are similar: 27% vs. 24.7% reduction of the error rate and 9.8% vs. 9.1% more reads mapped for the 76 cycle run and 45.6% vs. 45% reduction of the error rate and 49% vs. 44% more reads mapped for the 101 cycle run.

Figures and Tables

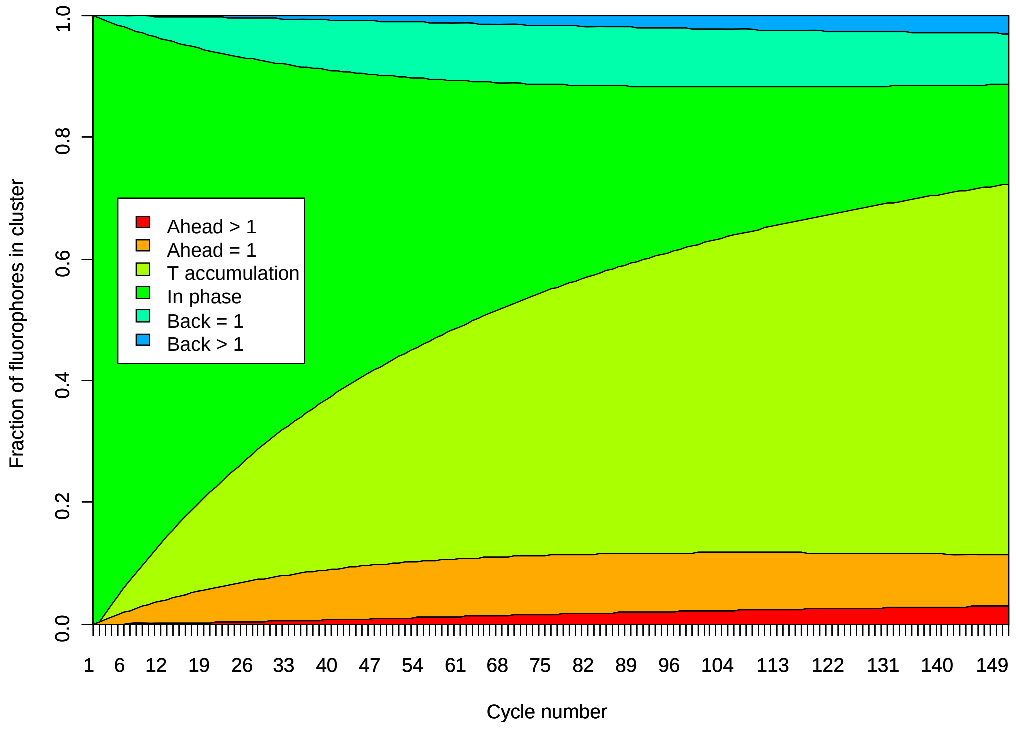

Figure S2: Development of the fraction of fluorophores representing the current cycle, the previous and the next cycles, cycles more than one ahead and more than one behind, as well as fluorophores attached due to T accumulation. The numbers are based on simulations of 10,000 clusters with a thousand identical sequences each. Sequences were created randomly, only using the GC content of PhiX 174 (44.7%) as reference. We used a model with pre-phasing (0.4% per cycle), phasing (0.4% per cycle) and T accumulation (3.8% per cycle) and simulated the fluorophores for 150 sequencing cycles.

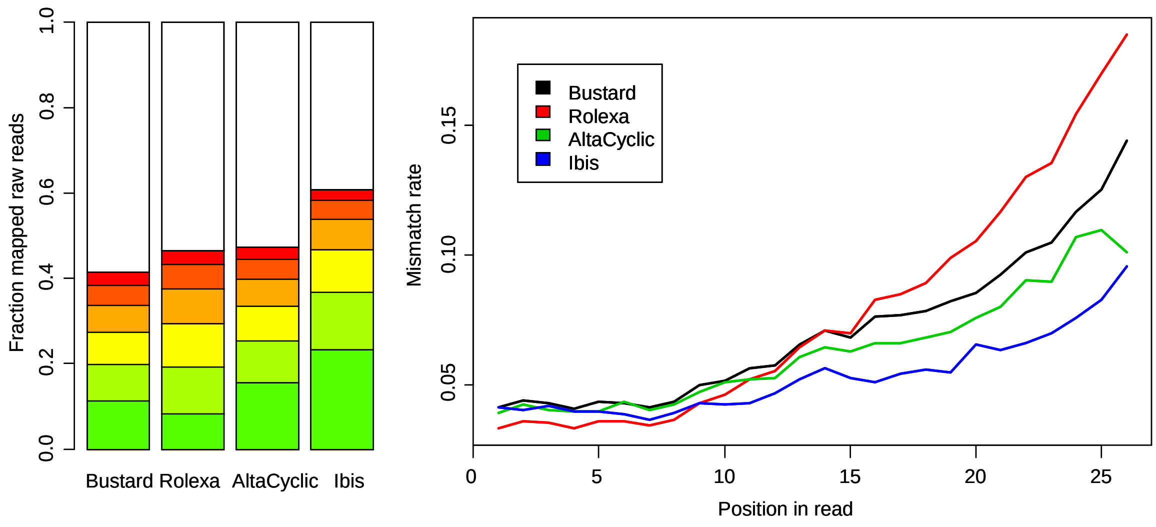

Figure S3: PhiX control lane of a 26 cycle Genome Analyzer I run

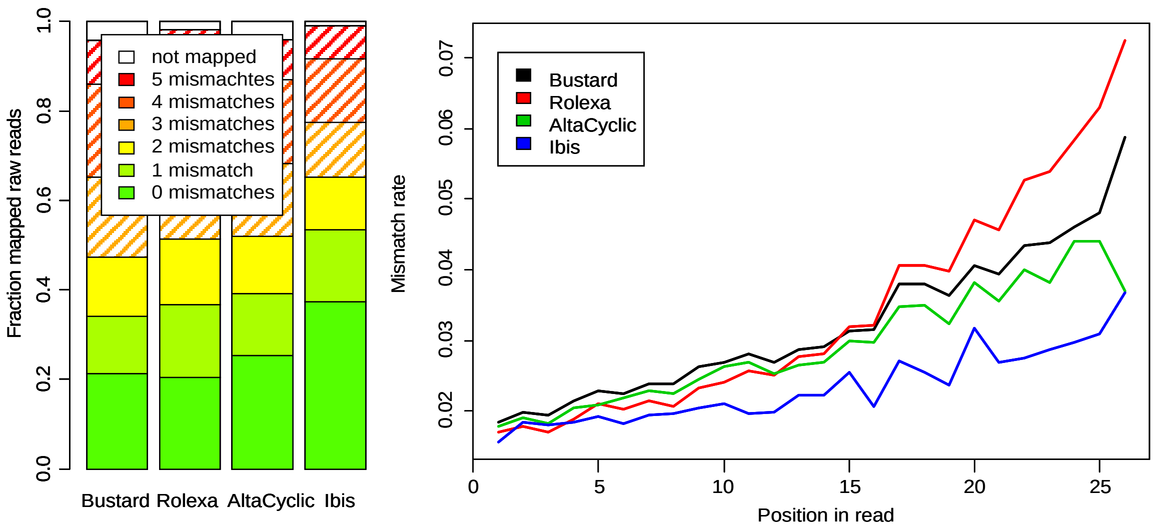

Figure S4: Human shotgun lane on a 26 cycle Genome Analyzer I run

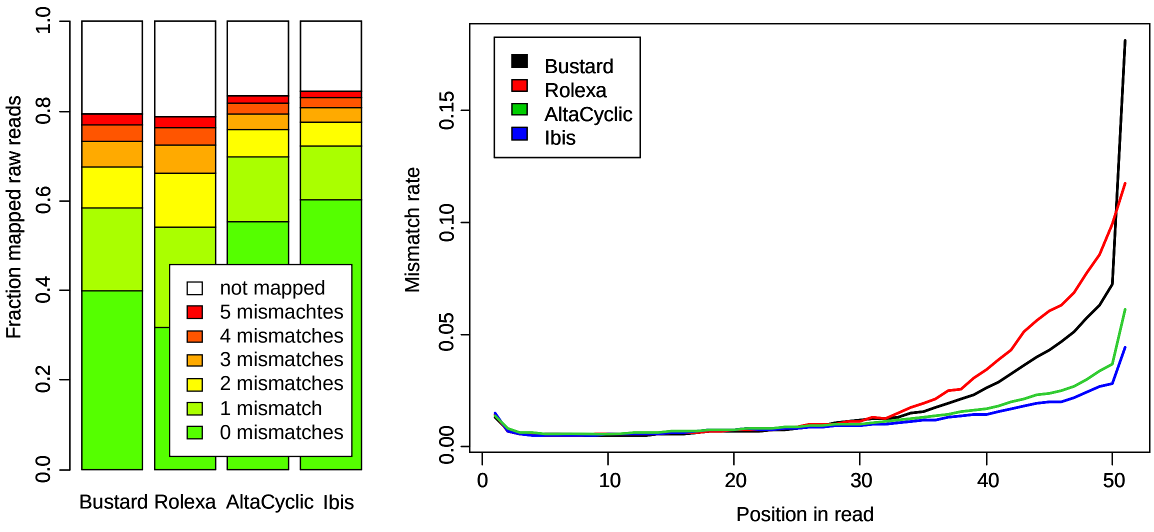

Figure S5: PhiX control lane of a 51 cycle Genome Analyzer II run (FC-104-100x chemistry)

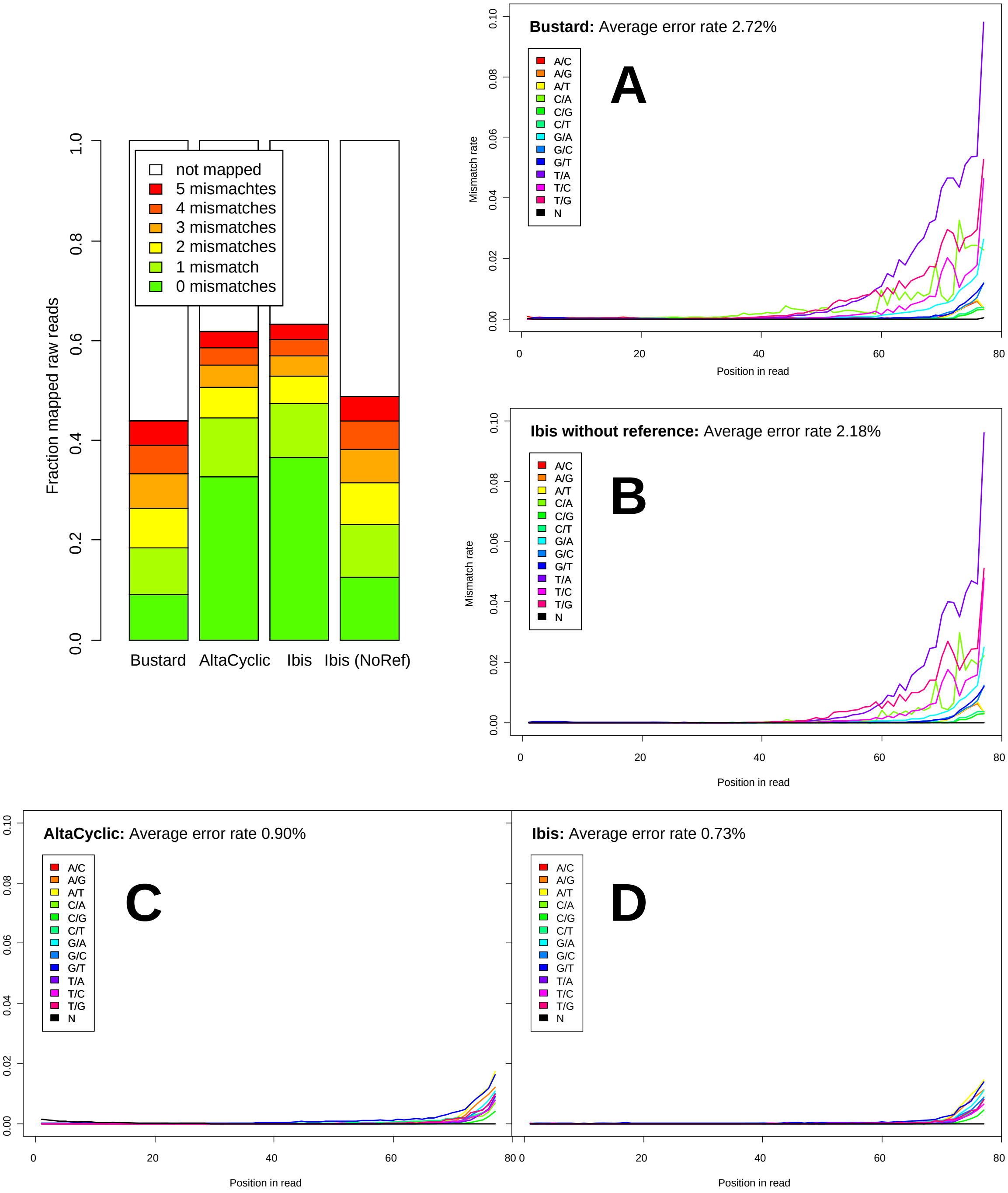

Figure S6: Mismatches to the reference sequence observed with different base calling strategies for the PhiX control lane of a 77 cycle Genome Analyzer II run with T accumulation (FC-204-20xx chemistry). Plot A shows the results for the standard Illumina base caller (Bustard), plots C (AltaCyclic) and D (Ibis) show strategies using a reference sequence (PhiX 174); in plot B Bustard reads have been used directly as input for the Ibis training process, without using a reference sequence.

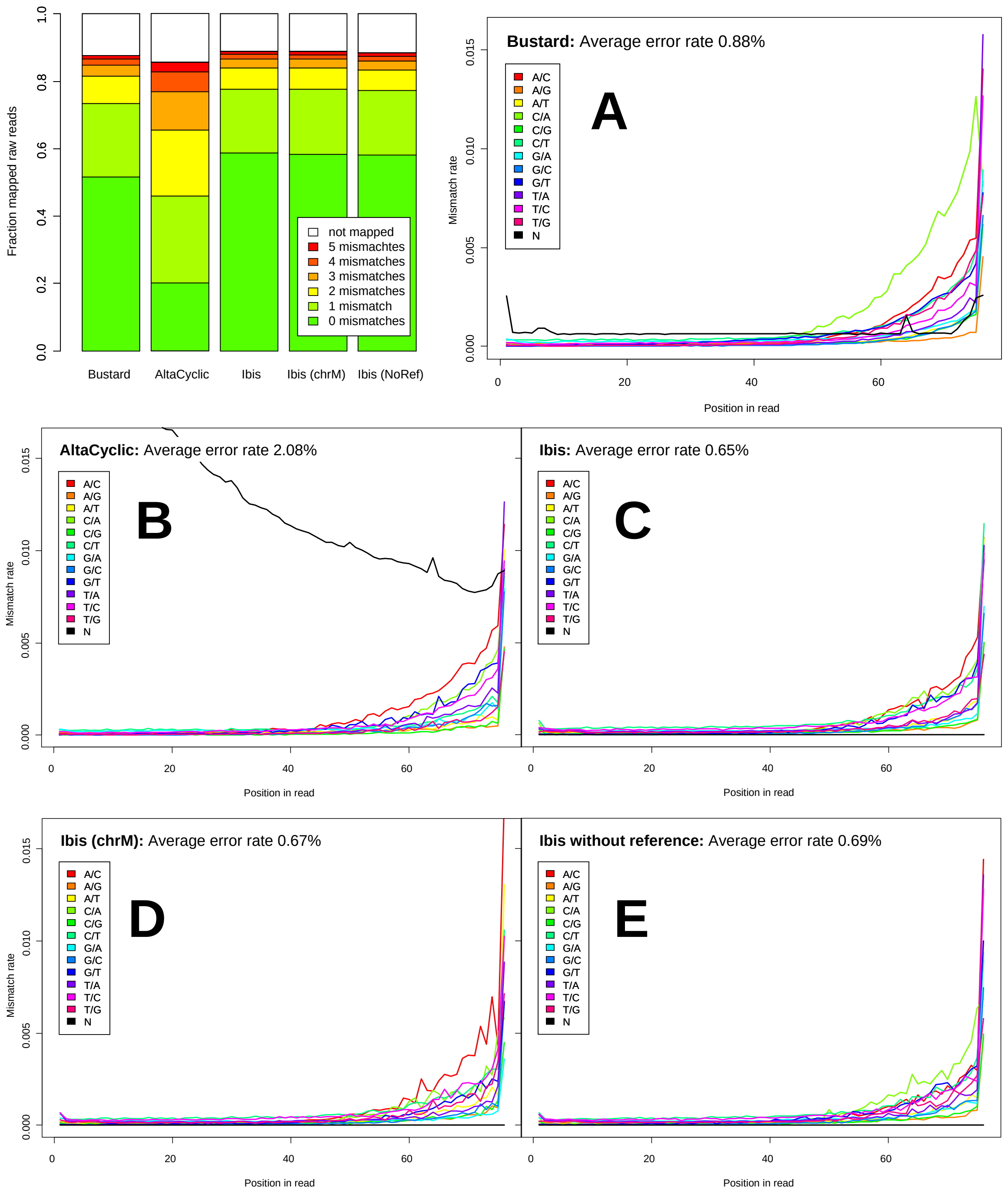

Figure S7: Mismatches to the reference sequence observed with different base calling strategies for the PhiX control lane of a 76 cycle Genome Analyzer II run using the current chemistry (FC-103-300x). Plot A shows the results for the standard Illumina base caller (Bustard). Plots B, C and D show strategies using a reference sequence (B and C PhiX 174 and D the human mitochondrial reference sequence). In plot E, Bustard reads have been used directly as input for the Ibis training process, without using a reference sequence.

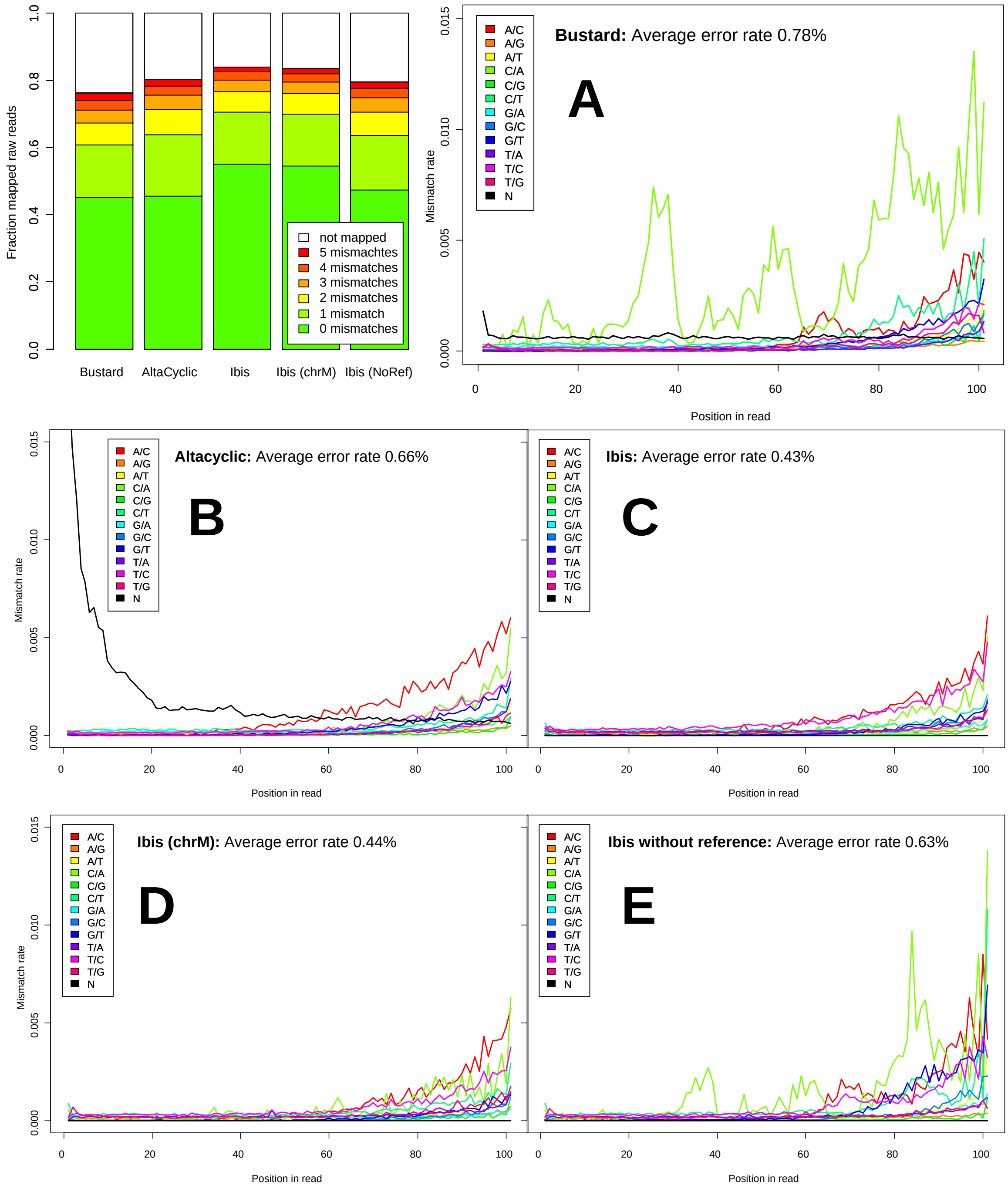

Figure S8: Mismatches to the reference sequence observed with different base calling strategies for the PhiX control lane of a 101 cycle Genome Analyzer II run using the current chemistry (FC-103-300x) and new polymerase (which will be released in the next version of Illumina sequencing kits). Plot A shows the results for the standard Illumina base caller (Bustard). Plots B, C and D show strategies using a reference sequence (B and C PhiX 174 and D the human mitochondrial reference sequence). In plot E, Bustard reads have been used directly as input for the Ibis training process, without using a reference sequence.

Table S2: Results of the simulation of 10,000 clusters, each with a thousand identical sequences. Random sequences were created using the GC content of PhiX 174 (44.7%) as a reference. We used a model with pre-phasing (0.4% per cycle), phasing (0.4% per cycle) and T accumulation (3.8% per cycle) and simulated the fluorophores attached for 150 sequencing cycles. For each cycle, we determined the number of fluorophores attached to the sequences of the cluster and calculated the fraction of fluorophores representing the current cycle, the previous and the next cycles as well as representing cycles more than one ahead and more than one behind. Further we calculated the fraction of fluorophores attached due to T accumulation. The results were averaged over all sequences. In the table T accumulation is given as a fraction of the starting fluorophores in a cluster.

Cycle	Back>1	Back=1	InPhase	Ahead=1	Ahead>1	T accumulation
1	0.0%	0.4%	99.2%	0.4%	0.0%	0.0%
2	0.0%	0.8%	98.4%	0.8%	0.0%	1.0%
3	0.0%	1.2%	97.6%	1.2%	0.0%	2.1%
4	0.0%	1.6%	96.9%	1.6%	0.0%	3.2%
5	0.0%	1.9%	96.1%	1.9%	0.0%	4.2%
6	0.0%	2.3%	95.4%	2.3%	0.0%	5.3%
7	0.0%	2.7%	94.6%	2.7%	0.0%	6.3%
8	0.0%	3.0%	93.9%	3.0%	0.0%	7.3%
9	0.1%	3.4%	93.1%	3.4%	0.1%	8.3%
10	0.1%	3.7%	92.4%	3.7%	0.1%	9.4%
11	0.1%	4.1%	91.7%	4.0%	0.1%	10.5%
12	0.1%	4.4%	91.0%	4.4%	0.1%	11.5%
13	0.1%	4.7%	90.3%	4.7%	0.1%	12.6%
14	0.1%	5.0%	89.7%	5.0%	0.1%	13.6%
15	0.2%	5.4%	89.0%	5.3%	0.2%	14.7%
16	0.2%	5.7%	88.3%	5.7%	0.2%	15.7%
17	0.2%	6.0%	87.7%	6.0%	0.2%	16.8%
18	0.2%	6.3%	87.0%	6.3%	0.2%	17.8%
19	0.2%	6.6%	86.4%	6.6%	0.2%	18.8%
20	0.3%	6.9%	85.7%	6.9%	0.3%	19.9%
21	0.3%	7.2%	85.1%	7.1%	0.3%	21.0%
22	0.3%	7.5%	84.5%	7.4%	0.3%	22.0%
23	0.4%	7.7%	83.9%	7.7%	0.3%	23.1%
24	0.4%	8.0%	83.3%	8.0%	0.4%	24.1%
25	0.4%	8.3%	82.7%	8.2%	0.4%	25.2%
26	0.4%	8.5%	82.1%	8.5%	0.4%	26.2%
27	0.5%	8.8%	81.5%	8.8%	0.5%	27.3%
28	0.5%	9.1%	80.9%	9.0%	0.5%	28.3%
29	0.5%	9.3%	80.3%	9.3%	0.5%	29.4%
30	0.6%	9.6%	79.8%	9.5%	0.6%	30.4%
31	0.6%	9.8%	79.2%	9.8%	0.6%	31.5%
32	0.7%	10.0%	78.6%	10.0%	0.6%	32.5%
33	0.7%	10.3%	78.1%	10.2%	0.7%	33.6%
34	0.7%	10.5%	77.6%	10.5%	0.7%	34.6%
35	0.8%	10.7%	77.0%	10.7%	0.8%	35.7%
36	0.8%	11.0%	76.5%	10.9%	0.8%	36.7%
37	0.8%	11.2%	76.0%	11.1%	0.8%	37.8%
38	0.9%	11.4%	75.5%	11.4%	0.9%	38.8%
39	0.9%	11.6%	75.0%	11.6%	0.9%	39.9%
40	1.0%	11.8%	74.4%	11.8%	1.0%	40.9%
41	1.0%	12.0%	73.9%	12.0%	1.0%	42.0%
42	1.1%	12.2%	73.4%	12.2%	1.1%	43.0%
43	1.1%	12.4%	73.0%	12.4%	1.1%	44.1%
44	1.2%	12.6%	72.5%	12.6%	1.1%	45.1%
45	1.2%	12.8%	72.0%	12.8%	1.2%	46.2%
46	1.2%	13.0%	71.5%	13.0%	1.2%	47.2%
47	1.3%	13.2%	71.1%	13.2%	1.3%	48.2%
48	1.3%	13.4%	70.6%	13.3%	1.3%	49.3%
49	1.4%	13.6%	70.1%	13.5%	1.4%	50.3%
50	1.4%	13.7%	69.7%	13.7%	1.4%	51.4%
51	1.5%	13.9%	69.2%	13.9%	1.5%	52.4%
52	1.5%	14.1%	68.8%	14.0%	1.5%	53.5%
53	1.6%	14.3%	68.4%	14.2%	1.6%	54.5%
54	1.6%	14.4%	67.9%	14.4%	1.6%	55.6%
55	1.7%	14.6%	67.5%	14.5%	1.7%	56.6%
56	1.7%	14.7%	67.1%	14.7%	1.7%	57.7%

Cycle	Back>1	Back=1	InPhase	Ahead=1	Ahead>1	T accumulation
57	1.8%	14.9%	66.7%	14.9%	1.8%	58.8%
58	1.9%	15.1%	66.2%	15.0%	1.8%	59.8%
59	1.9%	15.2%	65.8%	15.2%	1.9%	60.9%
60	2.0%	15.4%	65.4%	15.3%	1.9%	61.9%
61	2.0%	15.5%	65.0%	15.5%	2.0%	62.9%
62	2.1%	15.7%	64.6%	15.6%	2.0%	64.0%
63	2.1%	15.8%	64.2%	15.7%	2.1%	65.0%
64	2.2%	15.9%	63.9%	15.9%	2.2%	66.1%
65	2.2%	16.1%	63.5%	16.0%	2.2%	67.1%
66	2.3%	16.2%	63.1%	16.2%	2.3%	68.2%
67	2.3%	16.3%	62.7%	16.3%	2.3%	69.2%
68	2.4%	16.5%	62.4%	16.4%	2.4%	70.2%
69	2.5%	16.6%	62.0%	16.5%	2.4%	71.3%
70	2.5%	16.7%	61.6%	16.7%	2.5%	72.3%
71	2.6%	16.8%	61.3%	16.8%	2.6%	73.4%
72	2.6%	16.9%	60.9%	16.9%	2.6%	74.5%
73	2.7%	17.1%	60.6%	17.0%	2.7%	75.5%
74	2.7%	17.2%	60.2%	17.1%	2.7%	76.6%
75	2.8%	17.3%	59.9%	17.2%	2.8%	77.6%
76	2.9%	17.4%	59.5%	17.4%	2.8%	78.7%
77	2.9%	17.5%	59.2%	17.5%	2.9%	79.7%
78	3.0%	17.6%	58.9%	17.6%	3.0%	80.7%
79	3.0%	17.7%	58.5%	17.7%	3.0%	81.8%
80	3.1%	17.8%	58.2%	17.8%	3.1%	82.8%
81	3.2%	17.9%	57.9%	17.9%	3.1%	83.9%
82	3.2%	18.0%	57.6%	18.0%	3.2%	84.9%
83	3.3%	18.1%	57.2%	18.1%	3.3%	86.0%
84	3.3%	18.2%	56.9%	18.2%	3.3%	87.0%
85	3.4%	18.3%	56.6%	18.3%	3.4%	88.1%
86	3.5%	18.4%	56.3%	18.3%	3.4%	89.1%
87	3.5%	18.5%	56.0%	18.4%	3.5%	90.2%
88	3.6%	18.6%	55.7%	18.5%	3.6%	91.2%
89	3.7%	18.7%	55.4%	18.6%	3.6%	92.3%
90	3.7%	18.8%	55.1%	18.7%	3.7%	93.4%
91	3.8%	18.8%	54.8%	18.8%	3.8%	94.4%
92	3.8%	18.9%	54.5%	18.9%	3.8%	95.5%
93	3.9%	19.0%	54.3%	18.9%	3.9%	96.5%
94	4.0%	19.1%	54.0%	19.0%	3.9%	97.6%
95	4.0%	19.2%	53.7%	19.1%	4.0%	98.6%
96	4.1%	19.2%	53.4%	19.2%	4.1%	99.7%
97	4.2%	19.3%	53.2%	19.2%	4.1%	100.8%
98	4.2%	19.4%	52.9%	19.3%	4.2%	101.8%
99	4.3%	19.5%	52.6%	19.4%	4.2%	102.9%
100	4.3%	19.5%	52.4%	19.5%	4.3%	103.9%
101	4.4%	19.6%	52.1%	19.5%	4.4%	105.0%
102	4.5%	19.7%	51.8%	19.6%	4.4%	106.1%
103	4.5%	19.7%	51.6%	19.7%	4.5%	107.1%
104	4.6%	19.8%	51.3%	19.7%	4.6%	108.2%
105	4.7%	19.9%	51.1%	19.8%	4.6%	109.2%
106	4.7%	19.9%	50.8%	19.9%	4.7%	110.3%
107	4.8%	20.0%	50.6%	19.9%	4.7%	111.3%
108	4.8%	20.1%	50.3%	20.0%	4.8%	112.3%
109	4.9%	20.1%	50.1%	20.0%	4.9%	113.4%
110	5.0%	20.2%	49.8%	20.1%	4.9%	114.5%
111	5.0%	20.2%	49.6%	20.1%	5.0%	115.5%
112	5.1%	20.3%	49.4%	20.2%	5.1%	116.6%
113	5.2%	20.3%	49.1%	20.2%	5.1%	117.6%
114	5.2%	20.4%	48.9%	20.3%	5.2%	118.6%
115	5.3%	20.4%	48.7%	20.3%	5.3%	119.7%
116	5.4%	20.5%	48.5%	20.4%	5.3%	120.7%
117	5.4%	20.5%	48.2%	20.4%	5.4%	121.7%
118	5.5%	20.6%	48.0%	20.5%	5.4%	122.8%
119	5.5%	20.6%	47.8%	20.5%	5.5%	123.8%
120	5.6%	20.7%	47.6%	20.6%	5.6%	124.9%
121	5.7%	20.7%	47.4%	20.6%	5.6%	125.9%
122	5.7%	20.7%	47.2%	20.7%	5.7%	127.0%
123	5.8%	20.8%	46.9%	20.7%	5.7%	128.1%
124	5.9%	20.8%	46.7%	20.8%	5.8%	129.1%
125	5.9%	20.9%	46.5%	20.8%	5.9%	130.2%

Cycle	Back>1	Back=1	InPhase	Ahead=1	Ahead>1	T accumulation
126	6.0%	20.9%	46.3%	20.8%	5.9%	131.2%
127	6.0%	21.0%	46.1%	20.9%	6.0%	132.2%
128	6.1%	21.0%	45.9%	20.9%	6.1%	133.3%
129	6.2%	21.0%	45.7%	20.9%	6.1%	134.3%
130	6.2%	21.1%	45.5%	21.0%	6.2%	135.4%
131	6.3%	21.1%	45.3%	21.0%	6.3%	136.5%
132	6.4%	21.1%	45.1%	21.1%	6.3%	137.5%
133	6.4%	21.2%	44.9%	21.1%	6.4%	138.5%
134	6.5%	21.2%	44.7%	21.1%	6.4%	139.6%
135	6.6%	21.2%	44.6%	21.2%	6.5%	140.6%
136	6.6%	21.3%	44.4%	21.2%	6.6%	141.7%
137	6.7%	21.3%	44.2%	21.2%	6.6%	142.8%
138	6.7%	21.3%	44.0%	21.2%	6.7%	143.8%
139	6.8%	21.4%	43.8%	21.3%	6.8%	144.8%
140	6.9%	21.4%	43.6%	21.3%	6.8%	145.9%
141	6.9%	21.4%	43.5%	21.3%	6.9%	146.9%
142	7.0%	21.4%	43.3%	21.3%	6.9%	148.0%
143	7.1%	21.4%	43.1%	21.4%	7.0%	149.1%
144	7.1%	21.5%	42.9%	21.4%	7.1%	150.1%
145	7.2%	21.5%	42.8%	21.4%	7.1%	151.2%
146	7.2%	21.5%	42.6%	21.4%	7.2%	152.2%
147	7.3%	21.5%	42.4%	21.5%	7.2%	153.3%
148	7.4%	21.6%	42.3%	21.5%	7.3%	154.3%
149	7.4%	21.6%	42.1%	21.5%	7.4%	155.3%
150	7.5%	21.6%	41.9%	21.5%	7.4%	156.0%
